# Supplementary material for: Nutritional state-dependent modulation of insulin-producing cells in Drosophila
Source: eLife. 2025 Jan 29;13:RP98514. doi: 10.7554/eLife.98514 (PMC11778929; doi:10.7554/eLife.98514)
Supplement: Supplementary file 1. [file elife-98514-supp1.docx]

**Supplementary file 1a:** **p-values for statistical comparisons in Figure 2, activation window.**

Comparisons were made between the respective driver lines and Empty-split control flies. p-values were determined using the Wilcoxon rank-sum test. Columns 1-5 represent the five optogenetic activation cycles in Figure 2E, H and K. ‘Activation’ shows the p-values comparing average forward velocity pooled across all activation trials (Figure 2L).

| Genotype | 1 | 2 | 3 | 4 | 5 | Activation |
| --- | --- | --- | --- | --- | --- | --- |
| DILP2>CsChr, Fed | **0.0256** | 0.8694 | 0.5705 | 0.1867 | 0.1507 | **0.0133** |
| DILP2>CsChr, Starved | **3.4e^-09^** | **7.2e^-06^** | **1.2e^-06^** | **0.0021** | **0.0042** | **1.0e^-20^** |
| TDC2>CsChr, Fed | **2.1e^-05^** | **1.3e^-10^** | **7.7e^-12^** | **8.3e^-12^** | **3.6e^-12^** | **6.8e^-48^** |

**Supplementary file 1b:** **p-values for statistical comparisons in Figure 2, post activation window.**

P1-P5 represent the ‘post activation’ windows in Figure 2E, H and K. ‘Post’ contains the p-values comparing average forward velocity pooled across all trials (Figure 2M). Other details as for Supplementary file 1a.

| Genotype | P1 | P2 | P3 | P4 | P5 | Post |
| --- | --- | --- | --- | --- | --- | --- |
| DILP2>CsChr, Fed | 0.4357 | 0.9958 | 0.2543 | 0.7868 | 0.3874 | 0.6170 |
| DILP2>CsChr, Starved | **5.3e^-06^** | **0.0003** | **0.0008** | 0.2322 | 0.4540 | **1.7e^-8^** |
| TDC2>CsChr, Fed | **6.5e^-13^** | **1.7e^-13^** | **8.9e^-13^** | **4.7e^-13^** | **7.2e^-12^** | **7.3e^-58^** |

**Supplementary file 1c: p-values for statistical comparisons in Figure 4, activation window.**

1 - 5 represent the five activation cycles in Figure 4K and N. Other details as for Supplementary file 1a.

| Genotype | 1 | 2 | 3 | 4 | 5 |
| --- | --- | --- | --- | --- | --- |
| DH44>CsChr | **1.9e^-11^** | **2.1e^-12^** | **8.6e^-08^** | **4.5e^-06^** | **2.8e^-06^** |
| DH44^PI^>CsChr | **0.0001** | **0.0022** | 0.0540 | **0.0156** | **0.0015** |

**Supplementary file 1d: p-values for statistical comparisons in Figure 4, post activation window.**

P1-P5 represent the ‘post activation’ windows in Figure 4K and N. Other details as for Supplementary file 1a.

| Genotype | P1 | P2 | P3 | P4 | P5 |
| --- | --- | --- | --- | --- | --- |
| DH44>CsChr | **2.3e^-10^** | **2.7e^-13^** | **2.8e^-10^** | **4.0e^-08^** | **6.7e^-10^** |
| DH44^PI^>CsChr | 0.2711 | 0.1895 | 0.9044 | 0.0602 | **0.0054** |

**Supplementary file 1e: Number of IPCs and number of flies in each experiment**.

*The total number of flies and IPCs is not equal to the sum of all datasets because 19 IPCs from 19 flies were used in two figures: The ‘Pre’ datasets from Figure 3A, 3B and S4A were also used to establish the population averages in Figure 1D, and the ‘Fed’ dataset from Figure 1D was reused for ‘Mated’ and ‘3-6d’ datasets in Figure S2.

| **Dataset** | **Number of IPCs (N)** | **Number of flies** |
| --- | --- | --- |
| Figure 1D, Fed | 15 | 15 |
| Figure 1D, Starved | 23 | 19 |
| Figure 1G, 0.5-2h | 16 | 8 |
| Figure 1G, 6-8h | 10 | 7 |
| Figure 1G, HG, 3-5h | 7 | 4 |
| Figure 1G, HG, 6-12h | 11 | 9 |
| Figure 1G, HG, 18-24h | 11 | 9 |
| Figure 1H, HG+SD | 12 | 8 |
| Figure 1H, HF | 10 | 5 |
| Figure 1H, HA+SD | 10 | 5 |
| Figure 1H, SD | 12 | 7 |
| Figure 1H, HP | 11 | 9 |
| Figure 3A | 6 | 6 |
| Figure 3B | 7 | 7 |
| Figure 4C and 4D | 10 | 10 |
| Figure 4G and 4H | 8 | 5 |
| Figure S2A, Virgins | 10 | 9 |
| Figure S2A, Mated | 15 | 15 |
| Figure S2A, Male | 8 | 7 |
| Figure S2B, 3-6d | 15 | 15 |
| Figure S2B, 7-8d | 10 | 8 |
| Figure S2B, 10-11d | 14 | 8 |
| Figure S2B, 21-26d | 9 | 8 |
| Figure S4A | 6 | 6 |
| **Total** | **217*** | **160*** |

**Supplementary file 1f: Number of DH44^PI^Ns in Figure 3.**

*The total number of flies and DH44^PI^Ns is not equal to the sum of individual datasets because the ‘Pre’ datasets from Figure 3G and Figure S4F were also used to establish population averages in Figure 3F.

| **Dataset** | **Number of DH44^PI^Ns (N)** | **Number of flies** |
| --- | --- | --- |
| Figure 3F, Fed | 13 | 11 |
| Figure 3F, Starved | 11 | 10 |
| Figure 3G | 7 | 7 |
| Figure S4F | 6 | 6 |
| **Total** | **24*** | **21*** |
